# Exploring the Role of Clustered Mutations in Carcinogenesis and Their Potential Clinical Implications in Cancer

**DOI:** 10.3390/ijms25126744

**Published:** 2024-06-19

**Authors:** Yi Li, Rui Zhu, Jiaming Jin, Haochuan Guo, Jiaxi Zhang, Zhiheng He, Tingming Liang, Li Guo

**Affiliations:** 1 Jiangsu Key Laboratory for Molecular and Medical Biotechnology, School of Life Science, Nanjing Normal University, Nanjing 210023, China; 09210320@njnu.edu.cn (Y.L.); 09210313@njnu.edu.cn (R.Z.); 231212007@njnu.edu.cn (H.G.); 231202046@njnu.edu.cn (J.Z.); 2 State Key Laboratory of Organic Electronics and Information Displays, Institute of Advanced Materials (IAM), Nanjing University of Posts and Telecommunications, Nanjing 210023, China; 1222014424@njupt.edu.cn (J.J.); 1023173006@njupt.edu.cn (Z.H.)

**Keywords:** clustered mutations, carcinogenesis, prognostic markers, cancer

## Abstract

Abnormal cell proliferation and growth leading to cancer primarily result from cumulative genome mutations. Single gene mutations alone do not fully explain cancer onset and progression; instead, clustered mutations—simultaneous occurrences of multiple mutations—are considered to be pivotal in cancer development and advancement. These mutations can affect different genes and pathways, resulting in cells undergoing malignant transformation with multiple functional abnormalities. Clustered mutations influence cancer growth rates, metastatic potential, and drug treatment sensitivity. This summary highlights the various types and characteristics of clustered mutations to understand their associations with carcinogenesis and discusses their potential clinical significance in cancer. As a unique mutation type, clustered mutations may involve genomic instability, DNA repair mechanism defects, and environmental exposures, potentially correlating with responsiveness to immunotherapy. Understanding the characteristics and underlying processes of clustered mutations enhances our comprehension of carcinogenesis and cancer progression, providing new diagnostic and therapeutic approaches for cancer.

## 1. Introduction

Cancer, a complex disease characterized by malignant cell growth, arises from the accumulation of genetic mutations affecting the genes involved in cell cycle regulation, DNA repair, and tumor suppression. These mutations can disrupt normal cell growth and division controls, leading to uncontrolled proliferation and cancer formation. While the roles of individual gene mutations in carcinogenesis have been extensively studied, recent research underscores the significance of clustered mutations in cancer initiation and progression [1]. Clustered mutations, defined as the simultaneous occurrence of multiple mutations within a localized genome region, can impact multiple genes and biological pathways, resulting in various functional abnormalities within a single cancer cell [2,3]. These mutations profoundly influence cancer biology, with cancer genomes often containing clusters imprinted by different mutational processes. Due to their prevalence, clustered mutations may serve as novel biomarkers in clinical settings [4]. Understanding their role in carcinogenesis is crucial for unraveling cancer’s complexities and improving clinical outcomes.

This study aimed to explore the significance of clustered mutations in carcinogenesis, their implications for cancer diagnosis and treatment, and their role in cancer evolution. Additionally, it examined the associations between clustered mutations, clinical features, prognosis, and responses to immunotherapy across various cancer types. This comprehensive overview of the current understanding of clustered mutations in carcinogenesis highlights the potential impact of these findings on cancer research and clinical practice.

## 2. Overview of Clustered Mutations

### 2.1. Clustered Mutations: Accumulation of Single-Nucleotide Variations in a Given Region

Mutations are permanent, inheritable changes in DNA, including nucleotide base alterations and structural variations in chromosomes. They play crucial roles in regulating various cellular pathways by altering gene activity and driving cancer development. Generally, mutations are classified as point mutations or structural variations (SVs) based on their size and formation mechanisms [5]. Point mutations are heritable changes in small DNA segments (1–50 bps), including insertions and deletions (indels) and single-nucleotide variants (SNVs). Indels, involving the gain or loss of nucleotides spanning less than 50 bps, often result from polymerase slippage [6]. SNVs involve a single-base-pair substitution, categorized into transversions (purine to pyrimidine switch or vice versa) and transitions (purine to purine or pyrimidine to pyrimidine change). In somatic tissues, SNVs are associated with activation-induced cytidine deaminase/apolipoprotein B mRNA-editing enzyme catalytic polypeptide-like (AID/APOBEC)-mediated deamination [7]. SVs, larger genomic changes (>50 bps), include copy number variations (CNVs), inversions, insertions, translocations, and complex combinations of these variants. The improper repair of double-strand breaks (DSBs), caused by endogenous factors (e.g., reactive oxygen species) or exogenous factors (e.g., chemical mutagens or high-energy photons), as well as transposable elements (TEs) like retrotransposons, is the main cause of SVs [8,9,10,11,12,13]. The distribution of point mutations and SVs across the genome is not random. Evidence suggests that multiple simple nucleotide variations tend to accumulate [14,15,16,17,18], leading to clustered mutations. Clustered mutations have been subclassified into several types, including doublet- and multi-base substitutions (DBSs and MBSs) [19], diffuse hypermutations termed *omikli* [20], and longer-strand coordinated events termed *kataegis* [21]. Clusters of mutations at specific sites often have significant functional implications, contributing to heterogeneous mutation rates throughout the genome. Factors shaping this irregularity include the unique biophysical attributes of external cancer-causing agents, perturbations of innate cellular mechanisms, and extensive mutational occurrences signaling a loss of genomic stability [20,22,23,24]. Clustered mutations can arise from several mechanisms, such as dysfunction in DNA repair pathways, encounters with environmental mutagenic agents, and the activity of AID and APOBEC3 enzyme groups, which induce mutations through deamination [19,20,25,26,27].

### 2.2. The Classifications of Clustered Mutations

Traditionally, the distinguishing factors among the four clusters of characteristic mutations include variations in size, spatial configurations, and underlying causes of occurrence (Table 1). DBSs and MBSs, subsets of single-base substitutions (SBSs), refer to tandem doublet and multiple-base substitutions, respectively. In many cancer genomes, DBSs occur more frequently than random SBS adjacency would predict, suggesting that frequent singular mutagenic incidents are responsible for these adjacent base changes [19]. According to mutational signatures, DBSs are predominantly characterized by CC>TT and CC>AA mutations [19]. Various mechanisms precipitate these occurrences, including malfunctioning DNA repair mechanisms and exposure to external mutagenic agents [19,23,25]. Conversely, most MBSs result from factors such as tobacco smoke or ultraviolet (UV) light exposure [1]. *Omikli* (Greek for fog) represents another ubiquitous pattern of mutation clusters, involving two to three substitutions on single-stranded DNA (ssDNA) and accounting for a large proportion of clustered substitutions [1]. For instance, *omikli* comprised 37.2% of clustered substitutions across 2583 samples from the Pan-Cancer Analysis of Whole Genomes (PCAWG) project [1]. Additionally, they constituted over half of all drivers of clustered substitutions at 50.5%, while DBSs, *kataegis*, and other clustered occurrences contributed between 14% and 18% each [1]. *Omikli* mutations are more concentrated in early-replicating genomic regions and are common in cancers with stable microsatellites, suggesting that varied Mismatch Repair (MMR) efficiency across gene-dense areas leads to a higher frequency of *omikli* incidents within cancer-associated genes [20]. The characteristics of *omikli* include distinct mechanisms for short versus intermediate and long clusters, constrained APOBEC3A expression, and a strong correlation between un-clustered APOBEC3A mutation burden and *omikli* [20]. However, only 16.2% of *omikli* events match the APOBEC3 mutational pattern, indicating that multiple mechanisms, including exogenous factors like tobacco carcinogens and UV light, can give rise to *omikli* [1]. *Kataegis* (Greek for thunderstorm) also involves diffuse hypermutation but depends on longer ssDNA tracks than *omikli*, making *kataegic* events less prevalent [2,28,29]. Most *kataegis* are enriched around 10 kb of rearrangement breakpoints, with fewer events around 1 Mb or more than 1.5 Mb from a detected breakpoint. The characteristics of *kataegis* include a higher occurrence of C>T and C>G mutations, a preference for TpC mutation patterns, and continuity in sequencing reads, indicating origin from the same parental allele (in cis) and potential co-occurrence with significant genomic structural changes [25]. The APOBEC enzymes likely contributing to cancer mutagenesis are APOBEC3A and APOBEC3B, with a stronger association between APOBEC3B expression and the overall mutational burden across various cancers compared to APOBEC3A [30]. In cluster mutations, APOBEC3B plays significant roles in *omikli*, while *kataegis* heavily relies on APOBEC3A [20]. Growing research suggests that *kataegis* development stems from the repair of DSBs via homologous recombination (HR) or break-induced replication, exposing long ssDNA tracts [29,31,32] generated by multiple mutational processes. Additionally, 76.1% of *kataegic* occurrences display mutational signatures linked to AID/APOBEC3 deaminase activity [1]. Recently, extrachromosomal DNA (ecDNA)-associated *kataegis*, termed *kyklonas* (Greek for cyclone), was found in 31% of samples with circular ecDNA and highly open chromatin, facilitating interactions across vast genomic distances [33,34]. In ecDNA regions containing cancer-related genes, there is a pronounced increase in *kyklonic* episodes and the associated mutational load compared to ecDNA segments devoid of such genes. *Kyklonas* are typically situated approximately 750 kb from the closest breakpoint. It is improbable that *kyklonic* events arise from structural rearrangements during ecDNA development; instead, they predominantly exhibit characteristic APOBEC3 signature patterns. Further investigations are required to fully understand this type of hypermutation.

**Figure 1:**
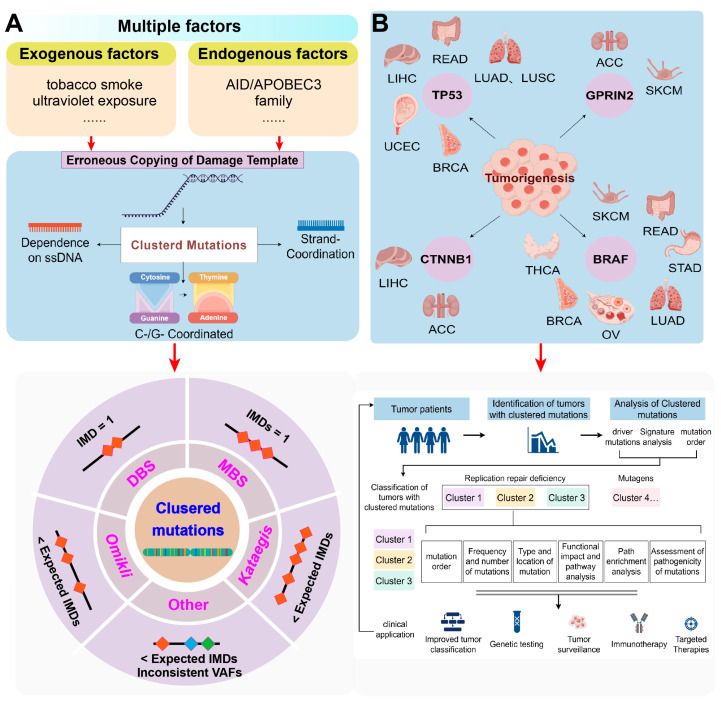
Clustered mutations and their potential role in cancer. (**A**). Biological genesis and the main types of cluster mutations. (**B**). Genes prone to clustered mutations in diverse cancers and clustered mutations with a crucial role in the development and progression of cancer. Understanding the functional consequences of clustered mutations is essential for unraveling the complex mechanisms underlying cancer and improving patient outcomes. Translating these research findings into clinical practice can ultimately enhance diagnostic, prognostic, and therapeutic decision-making for patients with cancer. ACC, adrenocortical carcinoma; BRCA, breast invasive carcinoma; LIHC, liver hepatocellular carcinoma; LUAD, lung adenocarcinoma; LUSC, lung squamous cell carcinoma; OV, ovarian serous cystadenocarcinoma; READ, rectum adenocarcinoma; SKCM, skin cutaneous melanoma; STAD, stomach adenocarcinoma; THCA, thyroid carcinoma; UCEC, uterine corpus endometrial carcinoma; IMD, inter-mutational distance; and VAFs, variant allele frequencies.

Researchers have utilized statistical approaches to clearly define and differentiate the four groups of clustered mutations based on various algorithms. For example, the inter-mutational distance (IMD) threshold can classify mutation types, and most methods also capture the highest disparity in variant allele frequencies (VAFs) or cancer cell fraction (CCF) to ensure that relevant mutations occur within neighboring cells [35]. Among clustered mutations with consistent VAFs or CCFs, those occurring in pairs with an IMD of one are classified as DBSs, while clusters of three or more adjacent mutations, each with an IMD of one, are classified as MBSs. Clusters consisting of two or three mutations below the sample-specific threshold and at least one mutation with an IMD greater than one are labeled as *omikli*, while similar clusters comprising four or more mutations fall under the category of *kataegis* [35]. All remaining clustered mutations with inconsistent VAFs or CCFs are categorized as a different class [35] (Figure 1A). This classification simplifies the segregation and categorization of clustered events, further enhancing the understanding of the characteristics and mutational mechanisms in cancer and normal somatic cells.

### 2.3. Main Features of Clustered Mutations

#### 2.3.1. Heterogeneity of Cluster Size

Several types of clustered mutations exhibit significant diversity across different organisms [3]. While SBSs involve only a few nucleotides, the cluster size can range from one to twenty kilobases. Remarkably, in *Escherichia coli*, proliferation in the presence of DNA-damaging agents can result in mutational clusters of up to 1500 kilobases [36].

#### 2.3.2. High Mutability in Specific Regions

Selected genes may acquire mutations more frequently in regions where genomic evolution in germlines or somatic cells favors increased variability [28]. A comparative analysis between human and chimpanzee genomic sequences, referencing an estimated shared progenitor, revealed a higher prevalence of mutation accumulation in segments designated as Human Accelerated Regions (HARs) throughout primate evolution [37]. Additionally, this clustering is related to the relaxation of stabilizing selection on mutations in duplicated genes and the local accumulation of unselected mutations [38].

However, on a broader scale, clustered mutations are relatively rare in the genome compared to individual mutations, with only 3.7% of all substitutions and 0.9% of all indels being clustered events. Despite their low frequency, mutagenesis clusters are significantly enriched among driver mutations. Clustered substitutions and indels contribute to 8.4% and 6.9% of driver mutations, respectively, indicating that these mutations play a critical role in cancer evolution.

#### 2.3.3. Accumulation of Fitness in Clusters

As discussed above, clustered mutations play crucial roles in various biological processes, particularly in carcinogenesis. While most individual mutations have negligible effects or reduce fitness, mutation clusters typically confer high fitness [39,40,41]. This characteristic allows these mutations to be selected and maintained during dramatic environmental changes, such as exposure to carcinogens. At the cellular level within cancer tissue, this high fitness enhances adaptability to the microenvironment, resulting in more aggressive cancer phenotypes [42].

#### 2.3.4. Strand Coordination between Mutation Types and Motifs

Another feature of clustered mutations is strand coordination between mutation types and motifs, indicating that they share the same strand and reference allele. The underlying biological causes for such strand coordination are partly linked to the unequal functions that the two DNA strands assume during various cellular activities [3]. Processes like replication and transcription necessitate DNA unwinding, exposing one or both strands. Crucially, upon disentanglement, one strand is often disproportionately revealed compared to its counterpart, fostering an uneven orchestration of mutations induced by APOBEC enzymes [43]. Thus, APOBEC3 patterns, which play a significant role in mutation clusters, are characterized by strand-coordinated mutations [41]. An investigation of 2583 cancer whole genomes from the PCAWG project revealed that approximately 81% of *kataegic* events are typically strand coordinated, indicative of damage or enzymatic changes on a single DNA strand.

#### 2.3.5. General Dependence on ssDNA

SsDNA lesions often evade repair via excision pathways due to the lack of a complementary template strand, increasing the risk of hypermutations. Lesions specific to ssDNA can similarly provoke hypermutation. During subsequent replication, these defects are frequently perpetuated as mutations through the involvement of error-prone translesion synthesis (TLS), leading to clusters of mutations [44]. Such mutation clusters within ssDNA are observed across various experimental frameworks, in the genetic sequences of cancers, and within ssRNA viruses [32,45,46]. Furthermore, hypermutations in ssDNA have also been found in the human germline [47].

#### 2.3.6. Preference toward C- or G-Coordinated Clusters

Studies indicate that clustered mutations exhibit a distinct preference for C>T or C>G base substitutions, likely due to APOBEC mediation [48]. While C>T transitions dominate in retroviral restriction, C>G alterations are equally prevalent in cancers [3]. In G-coordinated clusters, G>A variations occur more frequently than in other types [32]. Within the same system, C- or G-coordinated clustered mutations often appear in tCw motifs (where w corresponds to A or T), matching the signature of certain members of APOBEC family [2,49].

### 2.4. The Potential Mechanisms of Clustered Mutations

Mutation-clustering processes remain largely unidentified, but the potential origins of mutagenesis leading to multiple concurrent mutations have been linked to the erroneous copying of damaged templates by TLS polymerases [3,50]. One potential pathway involves intense mutagenic events causing random genome-wide damage that undergoes limited or no repair before double-strand DNA (dsDNA) replication. The replication stress response (RSR) primarily aims to preserve genomic integrity. During replication, high-fidelity DNA polymerases can be hindered by DNA damage, necessitating the use of low-fidelity TLS polymerases to continue polymerization over lesions [50]. Heightened TLS polymerase activity is frequently observed in cancerous tissues compared to non-cancerous tissues [51], indicating a significant role for these enzymes in cancer development. Ultimately, TLS DNA synthesis introduces multiple mutations in the nascent strand [52]. Damaged ssDNA without accurate repair is another source of clustered mutations. SsDNA lacks a template strand, making precise repair less probable. Lesions in ssDNA often result in mutation clusters due to the absence of a template or faulty repair mechanisms. Hypothetical mechanisms for ssDNA formation include R-loops [53], resection at specific DSBs, uncapped telomeres [28,54,55], break-induced replications (BIRs) [56], and chronic damage [3]. External or internal sources, such as UV radiation, methyl-methane sulfonate (MMS), hydrogen peroxide, sulfites, or human APOBEC3G cytidine deaminase, can cause structural DNA alterations, creating lasting ssDNA regions. However, ssDNA is unstable and prone to inducing multiple mutation clusters.

Recent studies have identified clustered mutations in circular ecDNA during carcinogenesis, occurring more frequently than in linear DNA. EcDNA mutation events are dominated by APOBEC3 patterns, characterized by strand-coordinated C>G and C>T mutations in the TpCpW context. EcDNA containing cancer-associated genes tends to experience repeated mutagenic attacks [14], forming mutation clusters more frequently, potentially driving cancer evolution.

## 3. Role of Clustered Mutations in Carcinogenesis

The arrangement of clustered mutations in cancer reveals the patterns and positions of mutations within cancer cell genomes, providing insights for improved patient care through refined diagnostics, prognostics, and personalized treatments. These mutations do not disperse randomly; instead, they aggregate closely due to genome structure and functionality [57]. The most extreme nonrandom distribution manifests as mutation clusters, densely packed in small genome regions, commonly observed in human cancer. The PCAWG initiative, examining the whole-genome sequences of 2583 cancer specimens, identified 1,686,013 single-base substitutions and 21,368 clustered indels [22]. Clustered substitutions comprised 45.7% DBSs, 0.7% MBSs, 37.2% *omikli*, and 7.0% *kataegis*, with notable variability within and among cancer types [1,22]. *Kataegis* was observed in 60.5% of all cancer cases within the PCAWG genome, particularly prevalent in lung squamous cell carcinoma, bladder cancer, acral melanoma, and sarcomas [22]. *Kataegis* typically involves C>N mutations in a TpC sequence context, likely due to APOBEC activity [2,23,58].

Exome-sequenced cancer analysis revealed strand-coordinated tCw mutations [49], indicating a preference for C or G coordination clusters in various cancer types [59]. Comprehensive examinations of whole-genome sequenced cancers detected tCw clusters in prostate, breast, and head-and-neck cancers, and among mutations from 2680 exome-sequenced cancers primarily sourced from The Cancer Genome Atlas (TCGA). This analysis expanded the spectrum of cancer types exhibiting these clusters to include cervical, lung adenocarcinoma, bladder, lung squamous cell carcinoma, ovarian, uterine endometrial, colorectal, rectal, kidney, and stomach cancers [49]. Cancer arises from numerous mutations disrupting normal cell growth and division control, with mutation-associated genes scattered across the genome. A prior investigation introduced Data-adaptive Mutation Clustering (DMCM) as a technique to identify cancer-associated mutation clusters [60]. DMCM uses kernel density estimation (KDE) with a data-adaptive bandwidth to estimate mutation density and identify clusters of varying lengths in amino acid sequences. By applying DMCM to over 500 mutated genes from 23 cancer types in TCGA, researchers pinpointed 1309 mutation clusters. Different cancer types exhibit distinct mutation cluster enrichment. Notably, *TP53* and *BRAF* mutations cluster in various cancer types, contributing to carcinogenesis. Somatic mutations in the *TP53* tumor suppressor gene are among the most common alterations in human cancers. The p53 protein’s role in inhibiting cell proliferation under stress and during senescence makes it a key target for inactivation in cancer [61]. Beyond single-base substitutions and allelic loss, *TP53* mutations show notable enrichment in clustered substitutions and indels [1]. *BRAF* oncogene mutations influence cancer cell proliferation, dissemination, and differentiation. The 600–601 cluster mutation in *BRAF* has been detected in diverse cancer types, such as BRCA, LUAD, OV, READ, STAD, and THCA [60].

Mutational signatures represent patterns resulting from internal and external factors influencing cancer formation, serving as historical indicators of cancer progression. These mechanisms encompass DNA damage, repair, and replication, potentially functioning normally or aberrantly. Unique mutational signatures arise from these processes, involving base substitutions, small indels, genome rearrangements, and chromosome copy number alterations [19]. Extensive studies have defined mutational signatures in 42 cancer types using 8836 samples, validating the connections between these signatures and clinical and genomic characteristics [62]. An analysis of clustered mutation distribution indicated a higher propensity for APOBEC- and Pol-η-related signatures (SBS9) and SBS40 to contribute to clustered mutations [25]. A detailed investigation into the relationship between mutational mechanisms and the cancer microenvironment revealed that signatures associated with APOBEC activity and mismatch repair deficits were positively linked to immune factors, whereas signatures attributed to aging showed inverse relationships. Moreover, the study indicated that certain signatures, such as SBS9, were beneficial, while others like SBS18 were detrimental to patient survival [62]. Research mapping mutation patterns at the gene level for 20 genes frequently altered in cancer revealed that signatures like SBS1 and SBS7 play major roles in mutations associated with cancer-driving genes (Table 2). Specifically, *TP53* and *KRAS* mutations are predominantly linked to SBS1 signatures. Additionally, an association between *PIK3CA*, *CTNNB1*, and the prevalence of SBS2 and SBS16 signatures was identified [63]. Significant research has delved into the varied mutation imprints found in cancer genomes among different malignancies, highlighting the crucial connection between mutation progression and the impact of environmental factors on various types of cancer.

Clustered mutations can induce chromosomal instability, facilitating oncogenesis by upregulating or silencing genes. This phenomenon involves chromosomal rearrangements, including translocations, losses, and amplifications. *Kataegis* regions often occur near sites of chromosomal disruption, such as breaks and rearrangements [2,23]. Research indicates that BIR, a specialized DNA replication process active during MMS exposure, frequently leads to mutation clusters in roughly half of repair events, linking these clusters with DNA breakage and rearrangements. Damage to BIR intermediates could be responsible for mutation clustering and the chromosomal changes observed in *kataegis* in human cancers [32]. BIR is a significant cause of mutation clusters due to damage in both yeast and cancer genomes. Variation in mutation cluster types within cancerous tissues may result from DNA impairment by APOBEC enzymes coupled with DNA cleavage from diverse damage forms or anomalies in checkpoint pathways in neoplastic cells [32,64].

Mutational clustering might play a role in the progression of cancer and other diseases. APOBEC family members, due to their affinity for ssDNA, can induce a cascade of mutations when ssDNA remains exposed [65,66]. These mutations exhibit a strand-concordant pattern, with numerous cytosines on a single DNA strand undergoing alteration. This distinctive APOBEC-induced mutation signature has been detected in a subset of cancers, evident from cytosine and complementary guanine strand-coordinated clusters across whole-genome sequencing datasets [2,23]. A recent study investigated the correlation between APOBEC signature mutations and potential cancer driver mutations [49]. Three criteria are used to identify potential cancer driver mutations: a Benjamini–Hochberg-corrected q value of less than 0.05 after CRAVAT analysis, inclusion in the COSMIC database, and mutations affecting a subset of genes in the Cancer Gene Census with missense or nonsense mutations [67,68]. APOBEC enzyme characteristic mutations were more prevalent as oncogenic drivers in cases with pronounced APOBEC activity compared to those without evident APOBEC mutation signatures, indicating a potential role in cancer development. The occurrence of clustered driver substitutions varied significantly across different cancers and genes. A minority of driver mutations corresponded with clustering in certain genes: *TP53* had 4.5%, *KRAS* 3.7%, and *PIK3CA* 2.2%. Conversely, a significant proportion of driver mutations formed clusters in other genes, with *BTG1* at 73.1%, *SGK1* at 66.6%, *EBF1* at 60.0%, and *NOTCH2* at 38.5% [22]. The proportion of particular clustered mutations associated with driver events differed significantly across genes. For instance, UV-light-induced DBSs represented 93% of *BRAF* driver mutations, while *omikli* constituted 63% of clustered drivers in *BTG1* and *kataegis* accounted for all clustered *NOTCH2* driver mutations. Clustered indel drivers showed that single-base pair indels made up nearly half at 48.7%. Examining individual genes revealed stark contrasts: clustered indel drivers were seldom seen in *TP53* (2.4%), but were markedly prevalent in *ALB* (76.6%) [1].

In cancer, clustered mutations primarily arise from damage to ssDNA, as evidenced by experiments in yeast and mutation analyses [2]. Enzymes from the AID/APOBEC family, which target ssDNA, are significant contributors to DNA damage from multiple sources, ultimately facilitating the formation of the mutation clusters observed in cancer [2,23,25,29,49,65,69,70,71]. Within a cellular environment, the presence of detrimental elements like APOBEC enzymes leads to the accumulation of ssDNA, crucial in generating mutation clusters. The emergence of ssDNA is often a byproduct of compromised replication forks and double-strand breaks, which can result from various cellular activities and scenarios, such as replication stress triggered by oncogenes [72].

In summary, clustered mutations in cancer can increase genetic instability, affect oncogenes or tumor suppressor genes, and impair DNA repair mechanisms. These mutations also promote heterogeneity within cancers, potentially leading to clonal evolution.

## 4. The Potential Values of Clustered Mutation in Cancer Diagnosis and Treatment

In the examination of human oncogenesis, clusters of mutations serve as a critical tool for deciphering mutational mechanisms [59,73]. Cluster analysis has revealed the APOBEC mutational pattern, characterized by transitions from tCw to tTw or tGw. This pattern has facilitated statistical analyses of the frequency of APOBEC-induced mutational events across various cancer types [49,74]. Investigations have pinpointed an enrichment of the APOBEC mutational signature within bladder, breast, cervical, head and neck, and lung cancers. This finding aligns with parallel analyses of mutation signatures conducted across diverse cancer types [25,75]. Even in cancers where APOBEC mutagenesis is not prevalent, clusters coordinated around C or G bases still exhibit APOBEC mutation patterns and are often found near breakpoints of genetic rearrangements [3]. Mutational clusters in genes like *TP53*, *EGFR*, and *BRAF*, which are linked to alterations in patient overall survival, are identifiable across various sequencing datasets, including targeted diagnostic panels like MSK-IMPACT, which bear clinical significance [1]. Identifying APOBEC enzymes within the complex array of mutational processes in cancer genomes is facilitated by examining the basic patterns of clustered mutation spectra.

Analyzing mutation clustering is crucial for pinpointing APOBEC cytidine deaminases as a primary mutational force in oncogenesis. Further research on clustered mutagenesis can provide valuable insights into genome maintenance, with significant implications for human health. Clustered mutations also hold potential clinical value in cancer diagnosis and prognosis (Figure 1B). Certain clustered mutations can serve as biomarkers for diagnosing specific cancer types. A principal component (PC) analysis of the standardized mutation pattern frequencies per cancer type showed that the variance attributable to clustered mutations is approximately three times that of un-clustered mutations within the first six principal components [17]. Even small quantities of clustered changes are informative markers that distinguish between cancer samples based on somatic mutational processes.

Clustered mutations significantly impact cancer patient survival, aiding in personalized treatment plans by guiding drug selection. Patients with cancers characterized by initial episodes of intense hypermutations, such as those involving POLE mutations, often exhibit increased survival prospects [76,77,78]. These cancers rapidly accumulate critical mutations, decreasing mutation efficiency and increasing treatment sensitivity. Conversely, cancers that develop hypermutation signatures at later stages may indicate a more aggressive disease progression or emerging chemotherapy resistance. Such mutational hallmarks are used to trace genetic origins when replication repair is deficient [79,80]. Additionally, accumulated mutations in oncogenic genomes serve as potent diagnostic tools for identifying underlying germline susceptibility. For instance, if a young patient’s tumor exhibits clustered mutations typical of late-stage outbreaks, genetic testing for conditional mismatch repair deficiency syndrome should be offered to their family members. Mutational clusters strongly present in driver genes imply a link between altered gene expression and survival outcomes. Studies have shown that specific clusters, especially in genes with established oncogenic roles, serve as markers for estimating patient life expectancy. For example, detecting clustered mutations in the melanoma-associated *BRAF* gene correlates with more favorable survival rates, while similar patterns in lung cancer’s prevalent *EGFR* gene indicate a decline in survival rates. Such prognostic differentiations become evident only upon identifying these clustered mutations, a process achievable with widely adopted clinical platforms [1]. Thus, clustered mutations represent a straightforward and precise biomarker for evaluating patient survival prognosis.

Mutations that truncate can deactivate tumor suppressor genes, while their function can be compromised by missense mutations occurring in critical regions. Unlike oncogene hotspot mutations, these missense mutations may not recur at specific loci, but tend to cluster within regions of mutational hotspots [81]. Identifying specific mutation clusters is crucial for tracing minimal residual disease (MRD) [82], which denotes the small number of cancerous cells that may persist after therapy. Detecting these mutations allows doctors to evaluate treatment efficacy and monitor for disease recurrence. Clustered mutations in cancer significantly impact treatment selection and the development of personalized medicine strategies. Utilizing mutation clusters to delineate mutation signatures proves superior for identifying signatures characteristic of specific cancer lineages compared to approaches focusing on individual cancer genomes or mutation fragments. For example, distinctive mutations corresponding to SBS4, SBS7, and SBS16 show a higher incidence in lung cancer, melanoma, and liver cancer, respectively, when recognized through cluster-based signature allocation as opposed to segment-based or sample-level identification. These findings highlight the enhanced reliability of mutation clusters in assigning mutation signatures to somatic mutations [63]. In clinical applications, next-generation sequencing (NGS) [83] enables the comprehensive profiling of genetic alterations in cancers, leading to more precise diagnoses and treatment selections [1]. Some clustered mutations are targetable, allowing for the use of drugs specifically designed to inhibit or target these mutations. Targeted therapies can be more effective and have fewer side effects compared to conventional chemotherapy. For example, *EGFR* [84] inhibitors in lung cancer and *BRAF* inhibitors in melanoma [85] have shown significant success in patients with specific mutations. Clustered mutations can also contribute to the creation of neoantigens, unique antigens that can activate the immune system. There is a correlation between clustered mutations and the effectiveness of immune checkpoint inhibitors, which have been known to result in lasting remission for some patients [86]. Hypermutation is found in about 1 in 20 childhood cancers and 1 in 6 adult cancers, often associated with replication repair defects and prolonged exposure to genotoxic substances, which can enhance the efficacy of immune checkpoint inhibitors. Identifying clustered mutations can help to predict the presence of neoantigens and guide the use of immunotherapy for individual patients.

Additionally, particular patterns of mutation clustering might indicate a cancer’s susceptibility or resistance to specific treatments. During cancer relapse, therapeutic agents may induce hypermutation events. The use of chemotherapy drugs, including alkylating agents or thiopurines, has been linked to the activation of DNA replication repair mechanisms [87,88]. Cancers exhibiting hypermutation demonstrate resistance to various treatments, including chemotherapy, due to their rapid evolutionary changes and enhanced capacity to render critical genes nonfunctional [88]. Establishing thresholds and profiles for mutational burden could guide the avoidance of superfluous therapies in favor of precision medicine or immunotherapy approaches [89]. Identifying these mutations allows doctors to predict a patient’s response to specific therapies and tailor their treatment accordingly. Several potential drug targets can be used to address cluster mutations in cancer treatment. Based on the mechanism and impact of cluster mutations, common targets include oncogenes [90], tumor suppressor genes [91], and DNA repair genes [92]. Additionally, many cancer-related signaling pathways, such as the PI3K-Akt-mTOR pathway [93] and the MAPK pathway [94], can become dysregulated due to clustered mutations. Drugs can be developed to target specific components of these pathways and disrupt their aberrant signaling.

Therefore, clustered mutations can serve as potential therapeutic targets in cancer treatment. Some mutations may be targetable with specific inhibitors or therapies, including targeted therapies or immunotherapies. Identifying these clustered mutations can help to guide treatment selection and the development of personalized medicine approaches.

## 5. Conclusions and Prospect

Clustered mutations, defined as multiple mutations located closely in the genome, often result from mutagens or DNA repair errors. These mutations can disrupt normal cellular processes, promote uncontrolled cell growth, increase treatment resistance, and drive cancer heterogeneity, highlighting their critical role in carcinogenesis. Understanding the patterns and locations of clustered mutations allows researchers to identify the key genes and pathways involved in cancer initiation, growth, metastasis, and treatment resistance. These mutations can serve as diagnostic and prognostic markers for specific cancer types or subtypes, aiding in the identification of potential therapeutic targets and improving treatment outcomes while minimizing off-target effects.

Clustered mutations in cancer present challenges due to genomic complexity, varying impact levels, and tumor heterogeneity. The distinction between driver and passenger mutations, which range from single0nucleotide variations to structural changes, complicates accurate detection and characterization. Tumor heterogeneity leads to diverse mutation profiles within different regions, hindering a comprehensive genomic landscape assessment. Future research should focus on experimental and computational approaches to systematically investigate the functional significance of clustered mutations. Integrating functional data with genomic profiles can provide a comprehensive understanding of their biological implications. Additionally, combining clustered mutation data with other molecular and clinical information can enhance our understanding of their clinical relevance.

## Figures and Tables

**Table 1:** Main classifications of clustered mutations.

Type	Cluster Size	Distribution	Features	Origin	IMD	Number of Adjacent Mutations	Refs.
DBSs	Tandem doublet substitutions	Random adjacency of SBSs	CC>TT or AA	(1) Dysfunction of DNA repair mechanisms; (2) exposure to mutagens present in the surroundings	1	2	[19,23,25,35]
MBSs	Multiple-base substitutions			Exogenous factors		>3	[1,35]
*Omikli*	Two to three substitutions on ssDNA	Prevalent in areas that reproduce at an early stage and frequently observed in cancers exhibiting consistent microsatellites	(1) Mechanism of short clusters distinct from intermediate and long clusters; (2) A3 expression frequently constrained; (3) strong correlation with burden of un-clustered A3 mutations	(1) APOBEC3 mutational patterns; (2) exogenous factors	<Expected value	2 or 3	[1,20,35]
*Kataegis*	Longer tracks of ssDNA than *omikli*	Enriched around 10 kb of rearrangement breakpoints	(1) C>T and C>G; (2) preference for TpC mutation patterns; (3) continuity in sequencing reads; (4) potential co-occurrence with significant genomic structural changes	Restoration of DNA integrity following the occurrence of dual-stranded DNA fractures or pathways involving replication induced by breaks		>4	[1,25,26,29,35]

**Table 2:** Mutation characteristics associated with cancer that are prone to clustered mutations.

Mutation Signatures	Mutation Type	Cancer
SBS6	DNA mismatch repair deficiency	Colorectal and uterine cancers
SBS15		
SBS10	Associated with a deficiency in POLE	Colorectal cancer
SBS7	Pertaining to UV exposure	Skin melanomas
SBS4	Associated with tobacco use	Lung cancer
SBS3	Lacking BRCA functionality	Breast and ovary cancers
SBS9	Associated with Pol-η activity	Hematologic cancer

## Data Availability

The data supporting the findings of this study can be obtained from the corresponding author upon reasonable request.

## References

[1] Bergstrom E.N., Luebeck J., Petljak M., Khandekar A., Barnes M., Zhang T., Steele C.D., Pillay N., Landi M.T., Bafna V. (2022). Mapping clustered mutations in cancer reveals APOBEC3 mutagenesis of ecDNA. Nature.

[2] Roberts S.A., Sterling J., Thompson C., Harris S., Mav D., Shah R., Klimczak L.J., Kryukov G.V., Malc E., Mieczkowski P.A. (2012). Clustered mutations in yeast and in human cancers can arise from damaged long single-strand DNA regions. Mol. Cell.

[3] Chan K., Gordenin D.A. (2015). Clusters of Multiple Mutations: Incidence and Molecular Mechanisms. Annu. Rev. Genet..

[4] Vural S., Wang X., Guda C. (2016). Classification of breast cancer patients using somatic mutation profiles and machine learning approaches. BMC Syst. Biol..

[5] Nesta A.V., Tafur D., Beck C.R. (2021). Hotspots of Human Mutation. Trends Genet..

[6] Montgomery S.B., Goode D.L., Kvikstad E., Albers C.A., Zhang Z.D., Mu X.J., Ananda G., Howie B., Karczewski K.J., Smith K.S. (2013). The origin, evolution, and functional impact of short insertion-deletion variants identified in 179 human genomes. Genome Res..

[7] Pecori R., Di Giorgio S., Paulo Lorenzo J., Nina Papavasiliou F. (2022). Functions and consequences of AID/APOBEC-mediated DNA and RNA deamination. Nat. Rev. Genet..

[8] Kumari S., Sharma S., Advani D., Khosla A., Kumar P., Ambasta R.K. (2022). Unboxing the molecular modalities of mutagens in cancer. Environ. Sci. Pollut. Res. Int..

[9] Savocco J., Piazza A. (2021). Recombination-mediated genome rearrangements. Curr. Opin. Genet. Dev..

[10] Kozmin S.G., Eot-Houllier G., Reynaud-Angelin A., Gasparutto D., Sage E. (2021). Dissecting Highly Mutagenic Processing of Complex Clustered DNA Damage in Yeast Saccharomyces cerevisiae. Cells.

[11] Srinivas U.S., Tan B.W.Q., Vellayappan B.A., Jeyasekharan A.D. (2019). ROS and the DNA damage response in cancer. Redox Biol..

[12] McKerrow W., Wang X., Mendez-Dorantes C., Mita P., Cao S., Grivainis M., Ding L., LaCava J., Burns K.H., Boeke J.D. (2022). LINE-1 expression in cancer correlates with p53 mutation, copy number alteration, and S phase checkpoint. Proc. Natl. Acad. Sci. USA.

[13] Giordani G., Cavaliere V., Gargiulo G., Lattanzi G., Andrenacci D. (2021). Retrotransposons Down- and Up-Regulation in Aging Somatic Tissues. Cells.

[14] Yi E., Gujar A.D., Guthrie M., Kim H., Zhao D., Johnson K.C., Amin S.B., Costa M.L., Yu Q., Das S. (2022). Live-Cell Imaging Shows Uneven Segregation of Extrachromosomal DNA Elements and Transcriptionally Active Extrachromosomal DNA Hubs in Cancer. Cancer Discov..

[15] Shi M.J., Meng X.Y., Fontugne J., Chen C.L., Radvanyi F., Bernard-Pierrot I. (2020). Identification of new driver and passenger mutations within APOBEC-induced hotspot mutations in bladder cancer. Genome Med..

[16] Rhee J.K., Yoo J., Kim K.R., Kim J., Lee Y.J., Chul Cho B., Kim T.M. (2019). Identification of Local Clusters of Mutation Hotspots in Cancer-Related Genes and Their Biological Relevance. IEEE/ACM Trans. Comput. Biol. Bioinform..

[17] Supek F., Lehner B. (2017). Clustered Mutation Signatures Reveal that Error-Prone DNA Repair Targets Mutations to Active Genes. Cell.

[18] Lawrence M.S., Stojanov P., Polak P., Kryukov G.V., Cibulskis K., Sivachenko A., Carter S.L., Stewart C., Mermel C.H., Roberts S.A. (2013). Mutational heterogeneity in cancer and the search for new cancer-associated genes. Nature.

[19] Alexandrov L.B., Kim J., Haradhvala N.J., Huang M.N., Tian Ng A.W., Wu Y., Boot A., Covington K.R., Gordenin D.A., Bergstrom E.N. (2020). The repertoire of mutational signatures in human cancer. Nature.

[20] Mas-Ponte D., Supek F. (2020). DNA mismatch repair promotes APOBEC3-mediated diffuse hypermutation in human cancers. Nat. Genet..

[21] D’Antonio M., Tamayo P., Mesirov J.P., Frazer K.A. (2016). Kataegis Expression Signature in Breast Cancer Is Associated with Late Onset, Better Prognosis, and Higher HER2 Levels. Cell Rep..

[22] Consortium I.T.P.-C.A.o.W.G. (2020). Pan-cancer analysis of whole genomes. Nature.

[23] Nik-Zainal S., Alexandrov L.B., Wedge D.C., Van Loo P., Greenman C.D., Raine K., Jones D., Hinton J., Marshall J., Stebbings L.A. (2012). Mutational processes molding the genomes of 21 breast cancers. Cell.

[24] Matsuda T., Kawanishi M., Yagi T., Matsui S., Takebe H. (1998). Specific tandem GG to TT base substitutions induced by acetaldehyde are due to intra-strand crosslinks between adjacent guanine bases. Nucleic Acids Res..

[25] Alexandrov L.B., Nik-Zainal S., Wedge D.C., Aparicio S.A., Behjati S., Biankin A.V., Bignell G.R., Bolli N., Borg A., Borresen-Dale A.L. (2013). Signatures of mutational processes in human cancer. Nature.

[26] Buisson R., Langenbucher A., Bowen D., Kwan E.E., Benes C.H., Zou L., Lawrence M.S. (2019). Passenger hotspot mutations in cancer driven by APOBEC3A and mesoscale genomic features. Science.

[27] Yu K. (2022). AID function in somatic hypermutation and class switch recombination. Acta Biochim. Biophys. Sin..

[28] Chan K., Roberts S.A., Klimczak L.J., Sterling J.F., Saini N., Malc E.P., Kim J., Kwiatkowski D.J., Fargo D.C., Mieczkowski P.A. (2015). An APOBEC3A hypermutation signature is distinguishable from the signature of background mutagenesis by APOBEC3B in human cancers. Nat. Genet..

[29] Taylor B.J., Nik-Zainal S., Wu Y.L., Stebbings L.A., Raine K., Campbell P.J., Rada C., Stratton M.R., Neuberger M.S. (2013). DNA deaminases induce break-associated mutation showers with implication of APOBEC3B and 3A in breast cancer kataegis. Elife.

[30] Hoopes J.I., Cortez L.M., Mertz T.M., Malc E.P., Mieczkowski P.A., Roberts S.A. (2016). APOBEC3A and APOBEC3B Preferentially Deaminate the Lagging Strand Template during DNA Replication. Cell Rep..

[31] DeWeerd R.A., Nemeth E., Poti A., Petryk N., Chen C.L., Hyrien O., Szuts D., Green A.M. (2022). Prospectively defined patterns of APOBEC3A mutagenesis are prevalent in human cancers. Cell Rep..

[32] Sakofsky C.J., Roberts S.A., Malc E., Mieczkowski P.A., Resnick M.A., Gordenin D.A., Malkova A. (2014). Break-induced replication is a source of mutation clusters underlying kataegis. Cell Rep..

[33] Peng Y., Li Y., Zhang W., ShangGuan Y., Xie T., Wang K., Qiu J., Pu W., Hu B., Zhang X. (2023). The characteristics of extrachromosomal circular DNA in patients with end-stage renal disease. Eur. J. Med. Res..

[34] Pongor L.S., Schultz C.W., Rinaldi L., Wangsa D., Redon C.E., Takahashi N., Fialkoff G., Desai P., Zhang Y., Burkett S. (2023). Extrachromosomal DNA Amplification Contributes to Small Cell Lung Cancer Heterogeneity and Is Associated with Worse Outcomes. Cancer Discov..

[35] Bergstrom E.N., Kundu M., Tbeileh N., Alexandrov L.B. (2022). Examining clustered somatic mutations with SigProfilerClusters. Bioinformatics.

[36] Parkhomchuk D., Amstislavskiy V., Soldatov A., Ogryzko V. (2009). Use of high throughput sequencing to observe genome dynamics at a single cell level. Proc. Natl. Acad. Sci. USA.

[37] Burbano H.A., Green R.E., Maricic T., Lalueza-Fox C., de la Rasilla M., Rosas A., Kelso J., Pollard K.S., Lachmann M., Pääbo S. (2012). Analysis of human accelerated DNA regions using archaic hominin genomes. PLoS ONE.

[38] Kusumi J., Ichinose M., Iizuka M. (2019). Effects of gene duplication, epistasis, recombination and gene conversion on the fixation time of compensatory mutations. J. Theor. Biol..

[39] Guthrie V.B., Allen J., Camps M., Karchin R. (2011). Network models of TEM beta-lactamase mutations coevolving under antibiotic selection show modular structure and anticipate evolutionary trajectories. PLoS Comput. Biol..

[40] Mannervik B., Runarsdottir A. (2010). The quest for molecular quasi-species in ligand-activity space and its application to directed enzyme evolution. FEBS Lett..

[41] Saona R., Kondrashov F.A., Khudiakova K.A. (2022). Relation Between the Number of Peaks and the Number of Reciprocal Sign Epistatic Interactions. Bull. Math. Biol..

[42] Liggett L.A., DeGregori J. (2017). Changing mutational and adaptive landscapes and the genesis of cancer. Biochim. Biophys. Acta Rev. Cancer.

[43] Sason I., Wojtowicz D., Robinson W., Leiserson M.D.M., Przytycka T.M., Sharan R. (2020). A Sticky Multinomial Mixture Model of Strand-Coordinated Mutational Processes in Cancer. iScience.

[44] Saini N., Gordenin D.A. (2020). Hypermutation in single-stranded DNA. DNA Repair.

[45] Bolli N., Avet-Loiseau H., Wedge D.C., Van Loo P., Alexandrov L.B., Martincorena I., Dawson K.J., Iorio F., Nik-Zainal S., Bignell G.R. (2014). Heterogeneity of genomic evolution and mutational profiles in multiple myeloma. Nat. Commun..

[46] Perelygina L., Chen M.H., Suppiah S., Adebayo A., Abernathy E., Dorsey M., Bercovitch L., Paris K., White K.P., Krol A. (2019). Infectious vaccine-derived rubella viruses emerge, persist, and evolve in cutaneous granulomas of children with primary immunodeficiencies. PLoS Pathog..

[47] Goldmann J.M., Seplyarskiy V.B., Wong W.S.W., Vilboux T., Neerincx P.B., Bodian D.L., Solomon B.D., Veltman J.A., Deeken J.F., Gilissen C. (2018). Germline de novo mutation clusters arise during oocyte aging in genomic regions with high double-strand-break incidence. Nat. Genet..

[48] Warren C.J., Santiago M.L., Pyeon D. (2022). APOBEC3: Friend or Foe in Human Papillomavirus Infection and Oncogenesis?. Annu. Rev. Virol..

[49] Roberts S.A., Lawrence M.S., Klimczak L.J., Grimm S.A., Fargo D., Stojanov P., Kiezun A., Kryukov G.V., Carter S.L., Saksena G. (2013). An APOBEC cytidine deaminase mutagenesis pattern is widespread in human cancers. Nat. Genet..

[50] Ghosal G., Chen J. (2013). DNA damage tolerance: A double-edged sword guarding the genome. Transl. Cancer Res..

[51] Albertella M.R., Lau A., O’Connor M.J. (2005). The overexpression of specialized DNA polymerases in cancer. DNA Repair.

[52] Twayana S., Bacolla A., Barreto-Galvez A., De-Paula R.B., Drosopoulos W.C., Kosiyatrakul S.T., Bouhassira E.E., Tainer J.A., Madireddy A., Schildkraut C.L. (2021). Translesion polymerase eta both facilitates DNA replication and promotes increased human genetic variation at common fragile sites. Proc. Natl. Acad. Sci. USA.

[53] Aguilera A., García-Muse T. (2012). R loops: From transcription byproducts to threats to genome stability. Mol. Cell.

[54] Mimitou E.P., Symington L.S. (2011). DNA end resection–Unraveling the tail. DNA Repair.

[55] Dewar J.M., Lydall D. (2012). Similarities and differences between “uncapped” telomeres and DNA double-strand breaks. Chromosoma.

[56] Liu L., Malkova A. (2022). Break-induced replication: Unraveling each step. Trends Genet..

[57] Rogozin I.B., Pavlov Y.I. (2003). Theoretical analysis of mutation hotspots and their DNA sequence context specificity. Mutat. Res..

[58] Nik-Zainal S., Van Loo P., Wedge D.C., Alexandrov L.B., Greenman C.D., Lau K.W., Raine K., Jones D., Marshall J., Ramakrishna M. (2012). The life history of 21 breast cancers. Cell.

[59] Roberts S.A., Gordenin D.A. (2014). Clustered and genome-wide transient mutagenesis in human cancers: Hypermutation without permanent mutators or loss of fitness. Bioessays.

[60] Lu X., Qian X., Li X., Miao Q., Peng S. (2019). DMCM: A Data-adaptive Mutation Clustering Method to identify cancer-related mutation clusters. Bioinformatics.

[61] Olivier M., Hollstein M., Hainaut P. (2010). TP53 mutations in human cancers: Origins, consequences, and clinical use. Cold Spring Harb. Perspect. Biol..

[62] Liao J., Bai J., Pan T., Zou H., Gao Y., Guo J., Xu Q., Xu J., Li Y., Li X. (2023). Clinical and genomic characterization of mutational signatures across human cancers. Int. J. Cancer.

[63] Lee S.Y., Wang H., Cho H.J., Xi R., Kim T.M. (2022). The shaping of cancer genomes with the regional impact of mutation processes. Exp. Mol. Med..

[64] Deem A., Barker K., Vanhulle K., Downing B., Vayl A., Malkova A. (2008). Defective break-induced replication leads to half-crossovers in Saccharomyces cerevisiae. Genetics.

[65] Chan K., Sterling J.F., Roberts S.A., Bhagwat A.S., Resnick M.A., Gordenin D.A. (2012). Base damage within single-strand DNA underlies in vivo hypermutability induced by a ubiquitous environmental agent. PLoS Genet..

[66] Chelico L., Pham P., Goodman M.F. (2009). Mechanisms of APOBEC3G-catalyzed processive deamination of deoxycytidine on single-stranded DNA. Nat. Struct. Mol. Biol..

[67] Douville C., Carter H., Kim R., Niknafs N., Diekhans M., Stenson P.D., Cooper D.N., Ryan M., Karchin R. (2013). CRAVAT: Cancer-related analysis of variants toolkit. Bioinformatics.

[68] Forbes S.A., Bhamra G., Bamford S., Dawson E., Kok C., Clements J., Menzies A., Teague J.W., Futreal P.A., Stratton M.R. (2008). The Catalogue of Somatic Mutations in Cancer (COSMIC). Curr. Protoc. Hum. Genet..

[69] Burns M.B., Lackey L., Carpenter M.A., Rathore A., Land A.M., Leonard B., Refsland E.W., Kotandeniya D., Tretyakova N., Nikas J.B. (2013). APOBEC3B is an enzymatic source of mutation in breast cancer. Nature.

[70] Lada A.G., Dhar A., Boissy R.J., Hirano M., Rubel A.A., Rogozin I.B., Pavlov Y.I. (2012). AID/APOBEC cytosine deaminase induces genome-wide kataegis. Biol. Direct.

[71] Lada A.G., Stepchenkova E.I., Waisertreiger I.S., Noskov V.N., Dhar A., Eudy J.D., Boissy R.J., Hirano M., Rogozin I.B., Pavlov Y.I. (2013). Genome-wide mutation avalanches induced in diploid yeast cells by a base analog or an APOBEC deaminase. PLoS Genet..

[72] Halazonetis T.D., Gorgoulis V.G., Bartek J. (2008). An oncogene-induced DNA damage model for cancer development. Science.

[73] Roberts S.A., Gordenin D.A. (2014). Hypermutation in human cancer genomes: Footprints and mechanisms. Nat. Rev. Cancer.

[74] Burns M.B., Temiz N.A., Harris R.S. (2013). Evidence for APOBEC3B mutagenesis in multiple human cancers. Nat. Genet..

[75] Alexandrov L.B., Nik-Zainal S., Wedge D.C., Campbell P.J., Stratton M.R. (2013). Deciphering signatures of mutational processes operative in human cancer. Cell Rep..

[76] Albertson T.M., Ogawa M., Bugni J.M., Hays L.E., Chen Y., Wang Y., Treuting P.M., Heddle J.A., Goldsby R.E., Preston B.D. (2009). DNA polymerase epsilon and delta proofreading suppress discrete mutator and cancer phenotypes in mice. Proc. Natl. Acad. Sci. USA.

[77] Daee D.L., Mertz T.M., Shcherbakova P.V. (2010). A cancer-associated DNA polymerase delta variant modeled in yeast causes a catastrophic increase in genomic instability. Proc. Natl. Acad. Sci. USA.

[78] Kane D.P., Shcherbakova P.V. (2014). A common cancer-associated DNA polymerase epsilon mutation causes an exceptionally strong mutator phenotype, indicating fidelity defects distinct from loss of proofreading. Cancer Res..

[79] Amayiri N., Tabori U., Campbell B., Bakry D., Aronson M., Durno C., Rakopoulos P., Malkin D., Qaddoumi I., Musharbash A. (2016). High frequency of mismatch repair deficiency among pediatric high grade gliomas in Jordan. Int. J. Cancer.

[80] Durno C.A., Sherman P.M., Aronson M., Malkin D., Hawkins C., Bakry D., Bouffet E., Gallinger S., Pollett A., Campbell B. (2015). Phenotypic and genotypic characterisation of biallelic mismatch repair deficiency (BMMR-D) syndrome. Eur. J. Cancer.

[81] Gao J., Chang M.T., Johnsen H.C., Gao S.P., Sylvester B.E., Sumer S.O., Zhang H., Solit D.B., Taylor B.S., Schultz N. (2017). 3D clusters of somatic mutations in cancer reveal numerous rare mutations as functional targets. Genome Med..

[82] Peng Y., Mei W., Ma K., Zeng C. (2021). Circulating Tumor DNA and Minimal Residual Disease (MRD) in Solid Tumors: Current Horizons and Future Perspectives. Front. Oncol..

[83] Morganti S., Tarantino P., Ferraro E., D’Amico P., Duso B.A., Curigliano G. (2019). Next Generation Sequencing (NGS): A Revolutionary Technology in Pharmacogenomics and Personalized Medicine in Cancer. Adv. Exp. Med. Biol..

[84] Zhang T., Joubert P., Ansari-Pour N., Zhao W., Hoang P.H., Lokanga R., Moye A.L., Rosenbaum J., Gonzalez-Perez A., Martinez-Jimenez F. (2021). Genomic and evolutionary classification of lung cancer in never smokers. Nat. Genet..

[85] Hayward N.K., Wilmott J.S., Waddell N., Johansson P.A., Field M.A., Nones K., Patch A.M., Kakavand H., Alexandrov L.B., Burke H. (2017). Whole-genome landscapes of major melanoma subtypes. Nature.

[86] Campbell B.B., Light N., Fabrizio D., Zatzman M., Fuligni F., de Borja R., Davidson S., Edwards M., Elvin J.A., Hodel K.P. (2017). Comprehensive Analysis of Hypermutation in Human Cancer. Cell.

[87] Nguyen S.A., Stechishin O.D., Luchman H.A., Lun X.Q., Senger D.L., Robbins S.M., Cairncross J.G., Weiss S. (2014). Novel MSH6 mutations in treatment-naive glioblastoma and anaplastic oligodendroglioma contribute to temozolomide resistance independently of MGMT promoter methylation. Clin. Cancer Res..

[88] Swann P.F., Waters T.R., Moulton D.C., Xu Y.Z., Zheng Q., Edwards M., Mace R. (1996). Role of postreplicative DNA mismatch repair in the cytotoxic action of thioguanine. Science.

[89] Topalian S.L., Taube J.M., Anders R.A., Pardoll D.M. (2016). Mechanism-driven biomarkers to guide immune checkpoint blockade in cancer therapy. Nat. Rev. Cancer.

[90] Hung K.L., Yost K.E., Xie L., Shi Q., Helmsauer K., Luebeck J., Schopflin R., Lange J.T., Chamorro Gonzalez R., Weiser N.E. (2021). ecDNA hubs drive cooperative intermolecular oncogene expression. Nature.

[91] Williams A.B., Schumacher B. (2016). p53 in the DNA-Damage-Repair Process. Cold Spring Harb. Perspect. Med..

[92] Huang L., Lang G.T., Liu Q., Shi J.X., Shao Z.M., Cao A.Y. (2021). A predictor of pathological complete response to neoadjuvant chemotherapy in triple-negative breast cancer patients with the DNA repair genes. Ann. Transl. Med..

[93] Sato Y., Yoshizato T., Shiraishi Y., Maekawa S., Okuno Y., Kamura T., Shimamura T., Sato-Otsubo A., Nagae G., Suzuki H. (2013). Integrated molecular analysis of clear-cell renal cell carcinoma. Nat. Genet..

[94] Matano M., Date S., Shimokawa M., Takano A., Fujii M., Ohta Y., Watanabe T., Kanai T., Sato T. (2015). Modeling colorectal cancer using CRISPR-Cas9-mediated engineering of human intestinal organoids. Nat. Med..

